# Supplementary material for: Time-of-Flight Three Dimensional Neutron Diffraction in Transmission Mode for Mapping Crystal Grain Structures
Source: Sci Rep. 2017 Aug 25;7:9561. doi: 10.1038/s41598-017-09717-w (PMC5572055; doi:10.1038/s41598-017-09717-w)
Supplement: Supplementary file 1 — Supplementary information [file 41598_2017_9717_MOESM1_ESM.pdf]

Supplementary Information - Time-of-Flight Three Dimensional Neutron Diffraction in Transmission Mode for Mapping Crystal Grain Structures

Alberto Cereser[1,2], Markus Strobl[2,3], Stephen Hall[4,2], Axel Steuwer[5,6], Ryoji Kiyanagi[7], Anton Tremsin[8], Erik Bergbäck Knudsen[1], Takenao Shinohara[7], Peter Willendrup[1], Alice Bastos da Silva Fanta[9], Srinivasan Iyengar[10,2], Peter Mahler Larsen[1], Takayasu Hanashima[11], Taketo Moyoshi[11], Peter M. Kadletz[12], Philip Krooß[13], Thomas Niendorf[13], Morten Sales[1], Wolfgang W. Schmahl[12], and Søren Schmidt[1,*]

[1]NEXMAP, Department of Physics, Technical University of Denmark, Kgs. Lyngby, 2800, Denmark
[2]European Spallation Source ESS AB, Lund, 22592, Sweden
[3]Niels Bohr Institute, University of Copenhagen, Copenhagen, 2100, Denmark
[4]Division of Solid Mechanics, Lund University, Lund, 22362, Sweden
[5]Nelson Mandela Metropolitan University, Port Elizabeth, 6031, South Africa
[6]University of Malta, Msida, MSD 2080, Malta
[7]J-PARC center, Japan Atomic Energy Agency, Tokai-mura, 319-1195, Japan
[8]Space Sciences Laboratory, University of California at Berkeley, Berkeley, California 94720, USA
[9]Center for Electron Nanoscopy, Technical University of Denmark, Kgs. Lyngby, 2800, Denmark
[10]Division of Materials Engineering, Lund University, Lund, 22362, Sweden
[11]Research Center for Neutron Science and Technology, CROSS, Tokai, Naka-gun 319-1106, Japan
[12]Applied Crystallography and Materials Science, Department of Earth and Environmental Sciences, Ludwig-Maximilians-Universität, München, 80333, Germany
[13]Institut für Werkstofftechnik (Materials Engineering), Universität Kassel, Kassel, 34125, Germany
[*]ssch@fysik.dtu.dk

ABSTRACT

Time-of-flight three-dimensional neutron diffraction (ToF 3DND) is a technique to reconstruct shape, juxtaposition and orientation of the grains composing polycrystalline materials using time-of-flight neutrons. This document provides additional information on the ToF 3DND technique.

S1 Shape comparison procedure

At a given projection, once the extinction spots are segmented it is necessary to find out which spots belong to the same grain. To do so, a criterion was developed to measure similarity between different spots. The approach, illustrated in Fig. S1, employs the morphological operations of erosion and dilation[1]. In a binary image, dilation adds pixels to the boundaries of shapes, while erosion removes pixels on shape boundaries. To compare two spots A and B (A being the one with the larger area), we superimposed their centers of mass and measured the angular distribution of the pixels of B that are inside the eroded perimeter of A, and of the pixels of A that are outside the dilated perimeter of B. If the angular regions where the spots differ are smaller than the chosen thresholds, the spots are considered similar and thus relative to the same grain.

S2 Curves fitting the extinction spots distribution

The crystallographic orientation of a given grain can be calculated (non uniquely, see Sec. *U and CUC* below) by fitting the distribution of the relative combined extinction spots in the $\omega\lambda$-space, where ω is the sample rotation angle and λ the wavelength relative to a combined extinction spot. As a starting point, let us consider Bragg's law

$$\lambda = 2d \sin\theta \tag{S1}$$

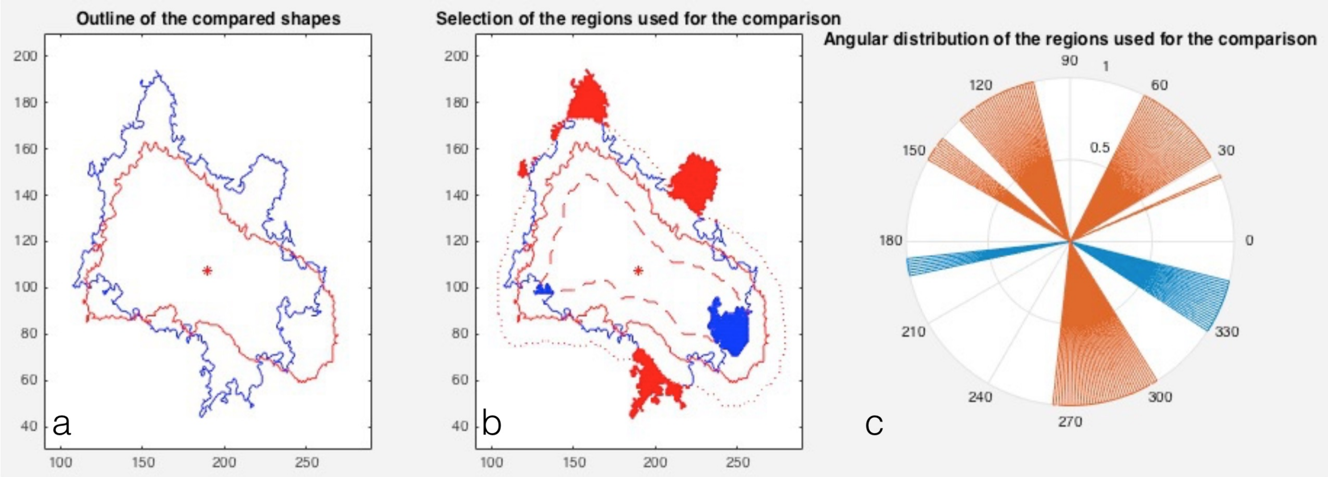

Figure S1. Criterion developed to measure how similar two extinction spots are. The procedure is used to check whether two extinction spots, registered at the same sample rotation angle, belong to the same grain. (**a**) Perimeter of the two extinction spots A (in blue) and B (in red) (area(A)>area(B)), translated such that they have the same centre of mass. (**b**) Selection of the regions used to measure how similar the two shapes are: portions of eroded B outside A (in blue), and portions of A outside dilated B (in red). (**c**) Angular distribution of the located regions. Considering the angular range over which the two types of portions are distributed, the developed algorithm decides whether two extinction spots are similar or not.

and the diffraction equation in the form used by Poulsen et al.[2]

$$\mathbf{G} = \frac{d}{2\pi}\Omega U \mathcal{B}\mathbf{h} \tag{S2}$$

where $|\mathbf{G}| = 1$, $d = \lambda/2\pi$ is the spacing between the lattice planes, Ω is the left-handed rotation matrix around the z-axis by an angle ω, U is the orientation matrix and $\mathcal{B}$ is the matrix which maps the hkl lattice, $\mathbf{h} = \begin{pmatrix} h \\ k \\ l \end{pmatrix}$, into reciprocal space. Eq. (S2) can be rewritten as

$$\mathbf{G} = \frac{\lambda}{4\pi}\Omega U \mathcal{B}\mathbf{h} \tag{S3}$$

Following the geometry sketched in Fig. 1, $\mathbf{G}$ can be expressed[3] as a function of the angles 2θ and η

$$\mathbf{G} = \frac{1}{2}\begin{pmatrix} \cos 2\theta - 1 \\ -\sin 2\theta \sin\eta \\ \sin 2\theta \sin\eta \end{pmatrix} \tag{S4}$$

Considering the first component of $\mathbf{G}$ and using Bragg's law, from Eq. (S4) one has

$$\mathbf{G}_1 = -\sin^2\theta = -\frac{\lambda^2}{4d^2} \tag{S5}$$

hence from Eq. (S3)

$$-\frac{\lambda^2}{4d^2} = \frac{\lambda}{4\pi}(\Omega U \mathcal{B}\mathbf{h})_1 \tag{S6}$$

Writing λ as a function of ω and introducing the vector $\mathbf{v} = \mathcal{B}\mathbf{h}$, one obtains the fundamental equation used to fit the point distribution in the $\omega\lambda$-space

$$\lambda(\omega) = -\frac{d^2}{\pi}(\Omega U \mathcal{B}\mathbf{h})_1 = -\frac{d^2}{\pi}(\Omega U \mathbf{v})_1 \tag{S7}$$

Renormalizing the scattering vector to $|\mathbf{G}| = \frac{\lambda}{2d}$ and considering the absolute value of Eq. (S3) leads to the expression $|\mathbf{G}| = \frac{\lambda}{2d} = \frac{\lambda}{4\pi}|\mathcal{B}\mathbf{h}|$. Using this relation and developing all terms, Eq. (S7) becomes

$$\begin{aligned}\lambda(\omega) = -\frac{4\pi}{|\mathcal{B}\mathbf{h}|^2}\big[&(u_{11}v_1 + u_{12}v_2 + u_{13}v_3)\cos\omega + \\ &+(u_{21}v_1 + u_{22}v_2 + u_{23}v_3)\sin\omega\big]\end{aligned} \tag{S8}$$

For a given reflection, corresponding to a given *hkl* family, the coefficients $A = u_{11}v_1 + u_{12}v_2 + u_{13}v_3$ and $B = u_{21}v_1 + u_{22}v_2 + u_{23}v_3$ have constant value, leading to the concise expression

$$\boxed{\lambda(\omega) = -\frac{4\pi}{|\mathcal{B}\mathbf{h}|^2}(A\cos\omega + B\sin\omega)} \tag{S9}$$

with the functions of ω being the only variables on the right-hand side.

S3 Grain indexing procedure

This is the recipe followed to index (non uniquely, see Sec. *U and CUC* below) a grain:

1. Scan the Rodrigues space for possible orientations[4]. For a cubic system, all orientations can be considered by sampling the points of the *fundamental zone*, a truncated cube with size $2\cdot(\sqrt{2}-1)$, whose corners are truncated by planes corresponding to rotations around (111) axes at a distance of $\tan(\pi/6)$ from the origin[5,6]. For each considered point (corresponding to an orientation), calculate the corresponding $\lambda(\omega)$ curves using Eq. (S9).
2. For each orientation, measure the distance of the fitting curves from the experimental values:
   (a) For each curve, measure the vertical distance from it (d_i) of the points located in a band around it (see Fig. S2).
   (b) Considering all the different $\lambda(\omega)$ curves, calculate $D = \sum_i d_i$.
3. Select the orientation whose $\lambda(\omega)$ curves return the minimum D.
4. Refine the selected orientation value by sampling, using a small grid, a region of the Rodrigues space built around the corresponding orientation vector.

For the Fe and the Co-Ni-Ga sample, grains are indexed considering the *hkl* families 110, 200 and 211. The forward model is applied to values with $\lambda > 2$Å: at lower wavelengths, the point distribution is more dense and harder to fit (see Fig. 5).

S4 U and CUC

For a given grain, fitting the distribution of its combined extinction spots using the forward model described in S3 does not uniquely determine its orientation. The ambiguity is derived in the following.

To be a proper rotation matrix, U must satisfy the condition $\det(U) = 1$, with

$$\det(U) = u_{11}(u_{22}u_{33} - u_{23}u_{32}) + u_{22}(u_{11}u_{33} - u_{13}u_{31}) + u_{33}(u_{11}u_{22} - u_{12}u_{21}) \tag{S10}$$

Considering the inversion $v_3 \to -v_3$, a change of sign of v_3 in Eq. (S8) results in the following changes:

1. $u_{13} \to -u_{13}$ and $u_{23} \to -u_{23}$, to conserve Eq. (S8).
2. $u_{31} \to -u_{31}$ and $u_{32} \to -u_{32}$, to conserve Eq. (S10).

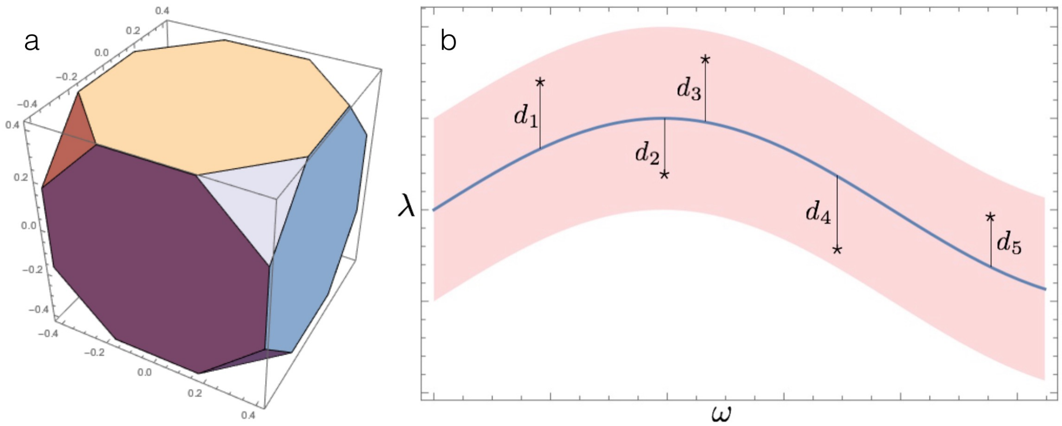

Figure S2. Details of the indexing procedure. The orientation of the grains is calculated using a forward model, sampling a region of the Rodrigues space, where orientations are represented as points, and selecting the orientation best fitting the point distribution in the $\omega\lambda$-space (see Fig. 5), where ω is the sample rotation angle and λ is the center of the wavelength interval where an extinction spot is recorded. (**a**) For a cubic system, all orientations can be considered by sampling the points of the *fundamental zone*, a truncated cube with size $2 \cdot (\sqrt{2} - 1)$, whose corners are truncated by planes corresponding to rotations around (111) axes at a distance of $\tan(\pi/6)$ from the origin[5,6]. (**b**) To calculate the distance of a curve from the experimental values, the sum of the vertical distances d_i is considered.

In other words, changing the sign of v_3 results in the following changes of sign for the elements of the orientation matrix U

$$U_{v_3} = \begin{pmatrix} + & + & + \\ + & + & + \\ + & + & + \end{pmatrix} \rightarrow U_{-v_3} = \begin{pmatrix} + & + & - \\ + & + & - \\ - & - & + \end{pmatrix} \tag{S11}$$

The two orientation matrices, U_{v_3} and U_{-v_3}, and the relative Rodrigues vectors, $\mathbf{r}_{v_3} = \begin{pmatrix} r_1 \\ r_2 \\ r_3 \end{pmatrix}$ and $\mathbf{r}_{-v_3} = \begin{pmatrix} -r_1 \\ -r_2 \\ r_3 \end{pmatrix}$, are related by the expressions

$$U_{-v_3} = CU_{v_3}C^{-1} \tag{S12}$$

$$\mathbf{r}_{-v_3} = C\mathbf{r}_{v_3} \tag{S13}$$

with

$$C = \begin{pmatrix} -1 & 0 & 0 \\ 0 & -1 & 0 \\ 0 & 0 & 1 \end{pmatrix} \tag{S14}$$

$$C = C^{-1} \tag{S15}$$

Consequently, based on transmission data alone the sign of the component of $\mathbf{G}$, v_3, parallel to the z-rotation axis cannot be determined. In other words, flipping the direction of all the diffracted neutrons along the z-axis (from upwards to downwards and vice versa) will result in the same distribution in the $\omega\lambda$-plane. This ambiguity can be resolved by either locating a diffraction spot, whose position uniquely determines v_3, or by introducing a second rotation axis perpendicular to the z-axis as part of the data acquisition procedure.

S5 Precision of grain boundary positions

The precision of the grain boundary positions was estimated by considering slices of a 3D grain map where, for a given grain, the value of each voxel corresponds to the number (later called *hits*) of extinction spots, relative to the given grain, that have

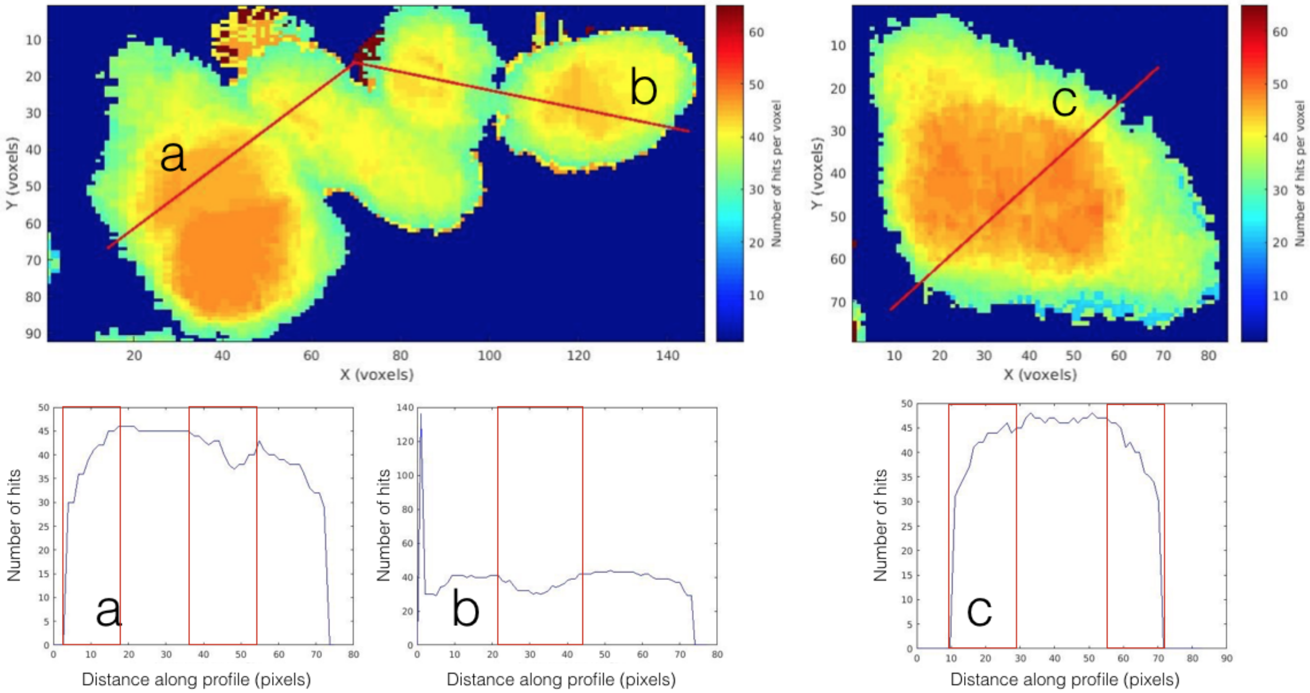

Figure S3. Details of the reconstruction slices used to estimate the precision of the grain boundary positions. For the the Fe sample, a 3D grain map was considered where, for a given grain, each voxel has a value defined by the number of extinction spots that are traced back to it (*hits*, shown in color scale). Due to higher mosaicity, the larger grain boundary positions uncertainty is expected for larger grains, whose extinction spots consistently have area larger than 1000 pixels. In the quantitative analysis, both isolated and neighboring grains were considered. For neighboring grains (i.e. *a* and *b*), the boundary accuracy is estimated as half the distance between two regions with high hits, each in a different grain. For an isolate grain (i.e. *c*), the accuracy is estimated as half the distance between the grain exterior and the high hits region in the grain. In the shown examples, distances are measured along the drawn lines.

been traced back to the voxel. In the Fe sample case, this hits-map was analyzed considering the larger grains, whose extinction spots have area consistently larger than 1000 pixels. Due to higher mosaicity, the extinction spots of these larger grains are recorded in more wavelength intervals than the spots associated with the smaller grains, which means that the boundaries are expected to be less well defined for the larger grains than for the smaller grains. In the quantitative analysis of the precision of the grain boundary positions, two types of larger grains were considered, as illustrated in Fig. S3: isolated grains and grains neighboring with other ones. For an isolated grain, the boundary uncertainty is estimated as half the distance between the outside of the grain and the region inside the grain with consistently high number of hits (see Fig. S3). For neighboring grains, the grain boundary uncertainty is estimated as half the distance between two regions with high number of hits, each inside a different grain. This quantification is considered conservative, as it takes into account the larger grains that have higher mosaicity and, thus, are likely less well defined by the current ToF 3DND approach. A more exact estimate of the grain boundary precision will be the topic of a forthcoming work.

S6 Grain maps of the Co-Ni-Ga sample

The algorithms developed to reconstruct, from the transmission data, the 3D shape of the grains and their orientation were validated by considering a Co-Ni-Ga sample, made of two single-crystal cubes, 4 mm in side. Fig. S4 shows two grain maps of the Co-Ni-Ga sample, obtained using ToF 3DND and EBSD.

S7 Comparison with X-rays

The ToF 3DND approach reported in the main text, with data acquired using a transmission detector only, is a generalization of the X-ray based direct-beam DCT technique (DCT-I) developed by Ludwig *et al.*[8] In the article presenting the DCT-I technique, a 550 microns diameter aluminum sample was investigated using monochromatic X-rays, and 8 grains with mosaicity lower than 0.05° were reconstructed. It was not possible to reconstruct sample grains with larger mosaicity (0.2-1°), either due to

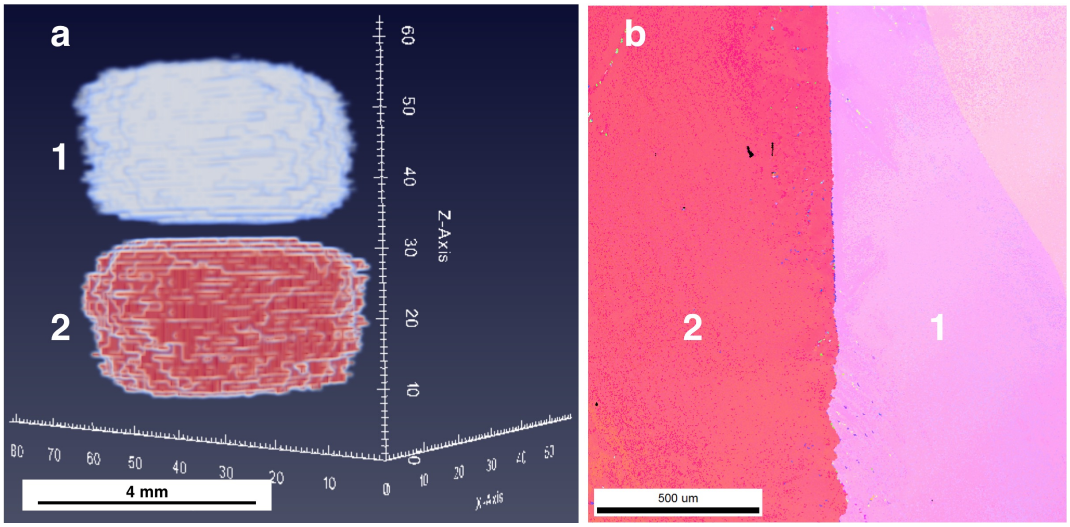

Figure S4. Grain maps of the Co-Ni-Ga sample studied at SENJU to validate the ToF 3DND indexing procedure. (**a**) ToF 3DND reconstruction of the two grains (cubes with 4 mm side) in the Co-Ni-Ga sample. The spacing between grains is due to cutoffs when back-projecting the extinction spots. (**b**) EBSD map of a sample slice, modified from Vollmer *et al.*[7] The grain numbering is the same in the two figures.

low signal-to-noise ratio or to overlapping signals. For this previous DCT-I case, 9000 projections were recorded with 0.02° integration steps (data acquired during sample rotation). In comparison, the ToF 3DND technique presented here utilized 48 projections with a static step size of 3° (sample static during acquisition). Each projection had 2423 wavelength images ranging from 0.4 Å to 4.4 Å. The grains in the 1 cm diameter iron sample had mosaicity up to 2° (based on EBSD measurements). 108 grains were reconstructed for this sample using the ToF 3DND filtering procedure, which is in good agreement with the 107 grains indexed in the far-field diffraction data (recorded by the detector banks on the SENJU instrument); this will be reported in a forthcoming manuscript. Therefore, the presented transmission mode ToF 3DND methodology can be considered as robust for a sample that cannot be studied by X-rays, due to low X-ray transmission.

References

1. Russ, J. C. *The image processing handbook* (CRC press, 2016).
2. Poulsen, H. F. and Nielsen, S. F. and Lauridsen, E. M. and Schmidt, S. and Suter, R. M. *et al.* Three-dimensional maps of grain boundaries and the stress state of individual grains in polycrystals and powders. *Journal of Applied Crystallography* **34**, 751–756 (2001).
3. Schmidt, S. Grainspotter: a fast and robust polycrystalline indexing algorithm. *Journal of Applied Crystallography* **47**, 276–284 (2014).
4. Morawiec, A. & Field, D. P. Rodrigues parameterization for orientation and misorientation distributions. *Philosophical Magazine A* **73**, 1113–1130 (1996).
5. He, Y. & Jonas, J. J. Representation of orientation relationships in Rodrigues–Frank space for any two classes of lattice. *Journal of Applied Crystallography* **40**, 559–569 (2007).
6. Kumar, A. & Dawson, P. R. Polycrystal plasticity modeling of bulk forming with finite elements over orientation space. *Computational Mechanics* **17**, 10–25 (1995).
7. Vollmer, M. *et al.* Damage evolution in pseudoelastic polycrystalline Co–Ni–Ga high-temperature shape memory alloys. *Journal of Alloys and Compounds* **633**, 288–295 (2015).
8. Ludwig, W., Schmidt, S., Lauridsen, E. M. & Poulsen, H. F. X-ray diffraction contrast tomography: a novel technique for three-dimensional grain mapping of polycrystals. I. Direct beam case. *Journal of Applied Crystallography* **41**, 302–309 (2008).
